# Emergence of multidrug-resistant and opportunistic fungal infections due to *Candida auris* in intensive care units

**DOI:** 10.22034/cmm.2024.345249.1558

**Published:** 2024-03-01

**Authors:** Peetam Singh, Anita Pandey

**Affiliations:** Department of Microbiology, Subharti Medical College, Meerut, Uttar Pradesh, India

**Keywords:** *Candida auris*, *Candida* infections, ICU, Healthcare-associated infections, Non-*albicans Candida*, Opportunistic pathogen

## Abstract

**Background and Purpose:**

An increasing number of invasive infections due to multidrug-resistant *Candida* species have been reported worldwide. Among these *Candida* species, *Candida auris* has attracted more attention in recent years due to major outbreaks in healthcare facilities globally and is considered an emerging pathogen. This study was planned to observe the occurrence of *C. auris* infections in a tertiary care hospital in India.

**Materials and Methods:**

The clinical specimens were inoculated on Sabouraud dextrose agar along with other conventional culture media for aerobic culture based on specimen type. The blood culture was performed by BacT/ALERT 3D automated blood culture system. After preliminary identification, the species-level identification of *Candida* was performed by the VITEK-2 compact automated system from bioMerieux, France.

**Results:**

Out of a total of 497 *Candida* isolates, 21.33% were *Candida albicans*, while 78.67% were non-*albicans Candida* species. *Candida auris* comprised 3.22% of all *Candida* isolates. Among various risk factors, intensive care unit stay was the most common risk factor associated with *C. auris* infection. The antifungal susceptibility data highlighted the resistance of *C. auris* against most of the antifungals except echinocandins.

**Conclusion:**

Infections due to *C. auris* are emerging in hospital environments, especially among individuals having various risk factors. The resistant nature of *C. auris* further complicates the situation leading to frequent antifungal treatment failure.

## Introduction

YEasts, including *Candida* species, are commensals on the skin, mucous membrane, gastrointestinal tract, and female genital tract. *Candida albicans* is considered the most common among clinically important *Candida* species. More than 50% of infections are caused by non-albicans Candida (NAC) species [ 1 ]. In recent years, an increasing number of invasive infections due to multidrug-resistant (MDR) *Candida* species have been reported worldwide. These emerging MDR *Candida* species include *C. glabrata*, *C. krusei*, *C. guilliiermondii* complex, *C. rugosa*, *C. lusitaniae*, *C. lipolytica*, *C. haemulonii* complex, *C. kefyr*, and *C. auris*. Among these potential MDR *Candida* species, *C. auris* has attracted more attention in recent years due to major outbreaks of invasive infections in healthcare facilities globally [ 1 , 2 ]. This is an emerging pathogen that is considered a threat to the global health system [ 3 ]. Following its first report in 2009 in Japan from a case of ear canal infection, *C. auris* has been reported worldwide including in India in various case reports, case series, and outbreaks in hospitals [ 4 - 9 ]. It has been found to be associated with various clinical conditions, including candidemia, urinary tract infections, pneumonia, and other invasive and non-invasive infections. Majority of the infections reported due to *C. auris* are among the patients with associated comorbidities or immunocompromised states. The patients admitted to intensive care units (ICU) or high-dependency units of the hospitals are at higher risk of getting infections with *C. auris* [ 5 - 9 ]. A resurgence of *C. auris* was reported among COVID-19 cases admitted to ICUs during the COVID-19 pandemic [ 10 , 11 ]. It is distinct from other *Candida* species due to its ability to acquire resistance to multiple antifungals and its propensity for transmission within a healthcare facility leading to outbreaks of hospital-acquired invasive infections associated with significant morbidity and mortality [ 12 , 13 ].

Reliable identification of *C. auris* using standard methods is difficult. The standard methods involve the panels of tests, including a combination of enzyme colorimetric tests, assimilation tests, and growth tests using carbohydrates and nitrogen compounds. Automated commercial identification systems rely upon disposable cards that contain sets of several conventional biochemical tests incorporated into microtiter wells used to identify a microorganism [ 14 ]. The reliable identification of *C. auris* can be carried out by matrix-assisted laser desorption ionization-time of flight mass spectrometry (MALDI-TOF MS) or various automated phenotypic identification systems, including VITEK-2 with updated database (version 8.01 or above) [ 14 , 15 ]. 

The data on *C. auris* infections is sparsely reported from India in case reports, case series, and a few outbreaks highlighting its clinical importance [ 4 - 9 ]. This study was planned to observe the occurrence of infections due to *C. auris* in a tertiary care medical teaching hospital in north India.

## Materials and Methods

This cross-sectional hospital-based observational study was conducted in the Microbiology Department of a tertiary care medical teaching institute in north India for a period of 18 months from November 2022 to April 2024. The clinical specimens of all types were collected from the patients as per the standard specimen collection guidelines following inclusion and exclusion criteria. Patients of all age groups and genders showing the growth of only *Candida* species on culture were included in the study. The patients with prior administration of antifungals and growth of microorganisms other than *Candida* species were excluded from the study. The samples showing the growth of multiple isolates were also excluded from the study.

### Institutional Ethics Committee Clearance

Approval of the institutional ethics committee was obtained before the commencement of the study with reference number SMC/UECM/2022/477B dated 14/10/2022.

### Sample Processing

**Figure 1:**
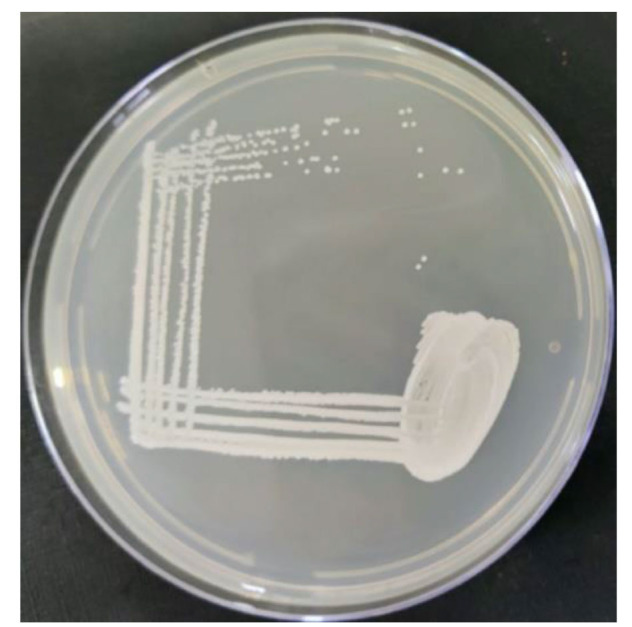
Colony characteristics of *Candida auris* on Sabouraud dextrose agar

**Figure 2:**
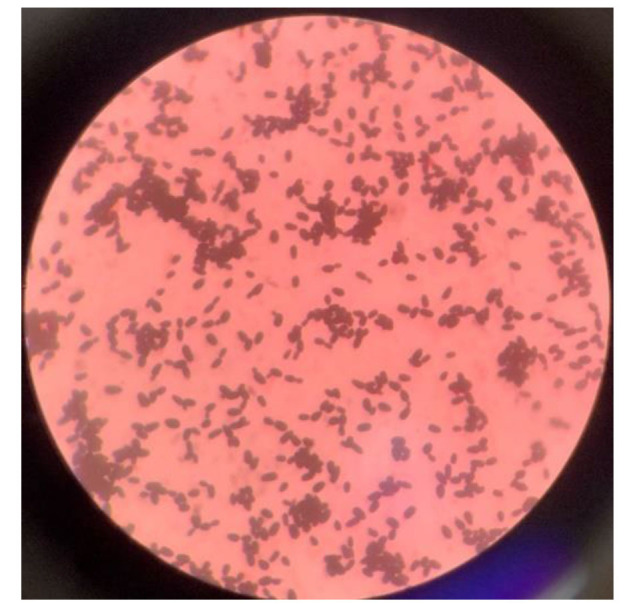
Microscopic features of *Candida auris* on Gram stain

**Figure 3:**
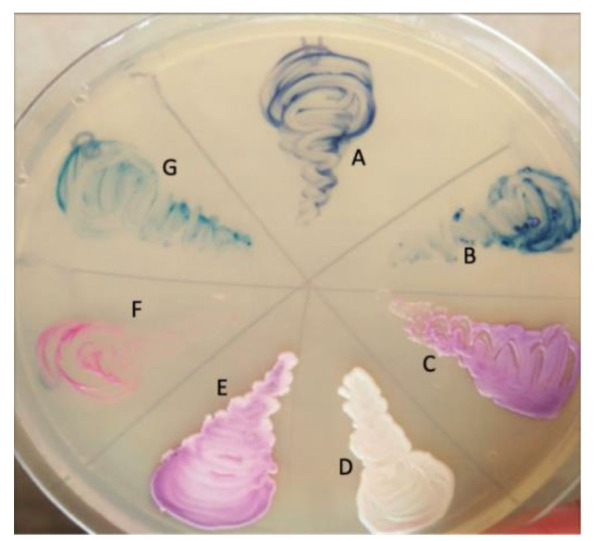
Characteristics of *Candida auris* on CHROMagar

The clinical specimens were inoculated on Sabouraud dextrose agar (SDA) along with other conventional culture media for aerobic culture based on specimen type except blood culture. The blood culture was performed by BacT/ ALERT 3D automated blood culture system (bioMerieux, France) using respective blood culture bottles for pediatric and adult patients procured from bioMerieux, France. After flagging positive by the system, the broth from blood culture bottles was sub-cultured on SDA, blood agar, chocolate agar, and MacConkey agar. Any growth on solid culture media was further processed for presumptive identification by conventional microbiological procedures, including colony morphology and Gram staining. After preliminary identification of *Candida*, the species level identification was carried out by growth characteristics on chromogenic agar *Candida* (CHROMagar) and germ tube test. The culture media and reagents were procured from HiMedia India Private Limited, India. The final species level identification was performed by VITEK-2 compact automated system (bioMerieux, France) by using YST and YS408 cards for identification of *Candida* species and determination of minimum inhibitory concentration (MIC) for antifungal susceptibility testing (AFST), respectively, procured from bioMerieux, France. The quality control (QC) strains of *Candida albicans* ATCC 14053 and *Candida parapsilosis* ATCC 22019 were used for QC of VITEK-2 YST card and VITEK-2 AST YS408 cards, respectively, as recommended by bioMerieux. The colony characteristics of SDA having smooth and white-to-cream-colored colonies are shown in Figure 1. The microscopic findings of gram-stained smear depicting Gram-positive yeast-like cells are shown in Figure 2. Characteristics of *C. albicans*, *C. tropicalis*, *C. parapsilosis*, *C. auris*, *C. kefyr*, *C. krusei*, and *C. dubliniensis* on CHROMagar showing pigment production by various *Candida* species are illustrated in Figure 3.

As antifungal susceptibility breakpoints for *C. auris* are not established, hence the breakpoints for antifungal susceptibility of *C. auris* are defined based on the breakpoints established on expert opinion for closely related *Candida* species. The correlation between microbiologic antifungal breakpoints for *C. auris* and clinical outcome is not known. The MIC-based antifungal susceptibility breakpoints for *C. auris* as suggested by the Centers for Disease Control and Prevention (CDC) for AFST of *C. auris* were used in this study. The MIC-based antifungal susceptibility breakpoints as suggested by CDC for interpretation of AFST of *C. auris* are shown in Table 1 [ 16 ].

**Table 1:** Interpretative breakpoints of Centers for Disease Control and Prevention of antifungals for *Candida auris*

Antifungal Name	Resistant Breakpoints (Minimum inhibitory concentration in µg/mL)
Fluconazole	≥32
Voriconazole	Based on susceptibility to fluconazole (surrogate marker for triazoles)
Amphotericin B	≥2
Caspofungin	≥2
Micafungin	≥4

### Statistical analysis

The data generated in this study was statistically analyzed in SPSS software (version 28.0, IBM Corp., Armonk, NY, USA). Categorical variables were compared using proportions and percentages. 

## Results

In total, 497 *Candida* isolates were isolated from the clinical samples collected during the study period. Out of the total *Candida* species, 106 (21.33%) were *C. albicans* while 391 (78.67%) were NAC species. Moreover, *C. auris* comprised 16 (3.22%) isolates of all *Candida* isolates and 4.09% of the NAC species.

Most of the *C. auris* isolates were recovered from adult patients (81.25%) followed by neonates (12.50%). Distribution of the demographic profile of the patients, including age, gender, and location, is summarized in Table 2. 

**Table 2:** Distribution of demographic characteristics of patients

Parameter		Number (N=16)
**Age**		
	Neonates (up to 1 month)	2 (12.50%)
	Infants (1 month-1 year)	0
	Children (1-12 year)	0
	Adolescents (13-17 years)	0
	Adults (18-65 years)	13 (81.25%)
	Elders (above 65 years)	1 (6.25%)
**Gender**		
	Male	12 (75%)
	Female	4 (25%)
**Location**		
	Intensive care unit	14 (87.50%)
	Surgical ward	2 (12.50%)

Among various risk factors, ICU stay was the most common risk factor associated with *C. auris* infection comprising 14 (87.50%) cases. A summary of all the cases, including various risk factors, type of clinical specimen, and treatment outcome associated with *C. auris* infection, is tabulated in Table 3. The clinical conditions associated with *C. auris* infection were bloodstream infection and catheter-associated urinary tract infection comprising 50% each as shown in Table 3. 

**Table 3:** Summary of cases and distribution of risk factors

Case No.	Age	Gender	Sample	Risk factors	Outcome
1	5 days	Male	Blood	ICU Stay, presence of CVC, extreme of age	Survived
2	6 days	Female	Blood	ICU Stay, presence of CVC, extreme of age	Deceased
3	23 years	Male	Blood	ICU Stay, chronic kidney disease, steroid therapy	Survived
4	24 years	Male	Blood	ICU Stay, steroid therapy	Survived
5	29 years	Male	Urine	ICU Stay, presence of CVC, prior antibiotic exposure, indwelling urinary catheter	Survived
6	29 years	Male	Blood	Surgery, prior antibiotic exposure, presence of CVC, indwelling urinary catheter, abdominal surgery	Survived
7	30 years	Male	Blood	Surgery, prior antibiotic exposure, presence of CVC, indwelling urinary catheter, steroid therapy	Survived
8	32 years	Male	Blood	ICU Stay, presence of CVC, indwelling urinary catheter, prior antibiotic exposure, steroid therapy	Survived
9	34 years	Male	Urine	ICU Stay, indwelling urinary catheter	Survived
10	49 years	Female	Urine	ICU Stay, indwelling urinary catheter, Diabetes mellitus	Survived
11	53 years	Female	Urine	ICU Stay, indwelling urinary catheter, presence of CVC, steroid therapy, Cushing syndrome with morbid obesity	Survived
12	54 years	Male	Urine	ICU Stay, indwelling urinary catheter, chronic kidney disease	Deceased
13	62 years	Female	Urine	ICU Stay, indwelling urinary catheter, Diabetes mellitus	Survived
14	76 years	Male	Urine	ICU Stay, indwelling urinary catheter, extreme of age, presence of CVC	Deceased
15	51 years	Male	Urine	ICU Stay, indwelling urinary catheter, Diabetes mellitus	Survived
16	41 years	Male	Blood	ICU Stay, presence of CVC, indwelling urinary catheter	Survived

ICU: Intensive care unit, CVC: Central venous catheter

**Figure 4:**
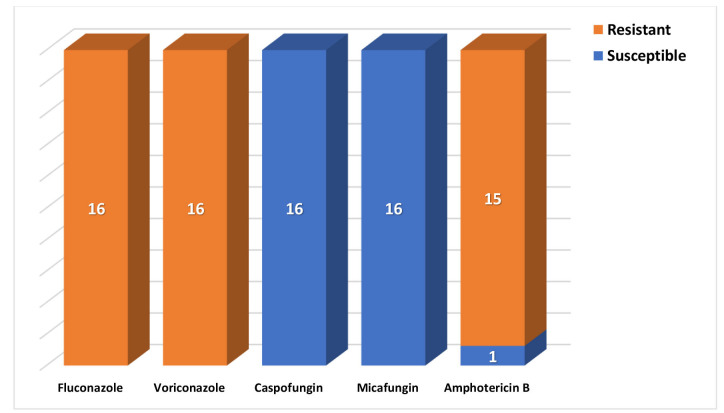
Antifungal susceptibility of *Candida auris* isolates

The AFST data revealed the MDR nature of all *C. auris* isolates, which were found sensitive to echinocandins only. The antifungal susceptibility results of *C. auris* against fluconazole, voriconazole, caspofungin, micafungin, and amphotericin B are depicted in Figure 4.

## Discussion

After the first report on the isolation of *C. auris* in 2009 in Japan from a case of ear canal infection, it has been reported worldwide, including in India, in various case reports, case series, and outbreaks in hospitals [ 4 - 9 ]. In the present study, there was a 3.22% prevalence of *C. auris* among all *Candida* species isolated from clinical samples during the study period. Studies on the prevalence of *C. auris* are rarely available as most of the data on *C. auris* are either in the form of case reports, case series, or outbreaks in ICUs. The reported data on *C. auris* infections is only the tip of the iceberg as the actual data may be many folds since the identification of *C. auris* is a major issue in routine microbiology laboratories due to a lack of reliable identification infrastructure. The conventional methods are not reliable for identification and frequently lead to misidentification of *C. auris* as other biochemically related species of *Candida*. The reliable identification of *C. auris* can be performed by MALDI-TOF MS or various automated phenotypic identification systems, including VITEK-2 with an updated database (version 8.01 or above) [ 4 , 15 ].

The gender-wise distribution showed that the majority of the cases were males (75%), compared to females (25%). The higher isolation rates from males may be due to differences in the genetic and constitutional make of individual genders. Most of the case reports and case series also go in favor of the predominance of males. Cases of *C. auris* infection consisted of bloodstream infection and catheter-associated urinary tract infection each comprising 50% of the present study. The major proportion of cases were from ICUs (87.50%), compared to post-operative surgical wards (12.50%). Moreover, ICU stay was the single most common risk factor associated with *C. auris* infection. All of the cases in this study were associated with multiple risk factors and the common risk factors were ICU stay in 87.50%, indwelling urinary catheter in 75%, presence of CVC in 56.25%, and prior antibiotic exposure in 25% of the cases, while extreme of age, steroid therapy, surgical procedures, chronic kidney disease, and diabetes mellitus were among less common risk factors. Almost similar risk factors were also observed in other studies, including a review on the epidemiology of *C. auris* in healthcare facilities in Asia by Thatchanamoorthy et al. [ 17 ]. These stated various risk factors correlated well with the outbreaks from all over the Asian countries. Similar risk factors were also documented in other studies highlighting the opportunistic nature of *C. auris* [ 3 , 5 , 6 , 9 - 13 ].

A mortality rate of 18.75% due to *C. auris* was observed in the present study. There are variations in the reported data on mortality due to *C. auris*, as no mortality was reported from seven cases reported in an outbreak investigation in south India by Sathyapalan et al. [ 10 ]. Higher rates of mortality due to *C. auris* were also reported, including the studies performed by Ortiz-Roa et al. and Kaki in 2023 comprising mortality rates of 38.1% and 66.7%, respectively [ 18 , 19 ]. The mortality due to *C. auris* infections is higher, compared to that due to other *Candida* species [ 18 ]. The mortality and morbidity depend on multiple factors, such as delayed diagnosis leading to delayed initiation of targeted antifungal treatment, immunocompromised states, and associated comorbidities. The MDR nature of *C. auris* in the scenario of misidentification as other *Candida* species also contributes to the higher morbidity and mortality due to treatment failure. The antifungal susceptibility data highlighted the resistance of *C. auris* against most of the antifungal drugs and its susceptibility only to echinocandins, such as caspofungin and micafungin. Only one isolate of *C. auris* showed susceptibility against amphotericin B. 

The limited available studies have highlighted the antifungal susceptibility data of *C. auris*. Almost similar findings were also observed in the studies performed by Prayag et al., Ashraf et al., and Sridharan et al. [ 20 - 22 ]. Since fluconazole is the most frequently used antifungal as an empirical medication in Indian hospitals, particularly in cases of unexplained fever or sepsis when the patient is not responding to antibiotics, this pattern of antifungal resistance is particularly concerning and disturbing. The echinocandins are advised as first-line antifungal treatment, subject to AFST due to the concern of azole and amphotericin B resistance in *C. auris* [ 21 , 23 ]. Reliable and accurate identification along with the AFST of *C. auris* is important for guiding therapy and evaluation of prognosis in ICUs, where MDR strains of the yeast are frequently prevalent [ 21 ]. If appropriate antifungal therapy is not timely instituted in conjunction with other interventions, such as the removal of invasive colonizing devices; invasive infections due to *C. auris* will likely result in a higher fatality rate [ 24 ].

Healthcare personnel in acute care settings have to use standard contact precautions to decrease the risk of transmission. Due to the MDR nature of *C. auris* isolates, as well as their ease of transmissibility in healthcare settings, it is imperative to quickly identify nosocomial outbreaks for the timely implementation of control measures. Since *C. auris* can live on a variety of surfaces, appropriate cleaning and disinfection techniques may be able to lower the risk of transmission [ 21 , 25 ]. As hand hygiene is the basic component of infection control, healthcare personnel should follow standard hand hygiene precautions to control the spread. The alcohol-based hand rubs are effective, as are chlorhexidine hand rubs against C. auris when hands are not visibly soiled. The contact precautions with gloves and gowns should be observed but gloves are not a substitute for hand hygiene [ 26 - 28 ].

There were certain limitations in this study. As this study was limited to a single hospital catering a a small proportion of the population, the findings cannot be generalized to the entire population. Moreover, molecular characterization of identification, including sequencing and genetic basis of antifungal drug resistance, was not studied due to limited resources.

## Conclusion

Infections due to *C. auris* are emerging in hospital environments, especially among individuals having various risk factors, such as ICU stay, presence of indwelling devices, and immunocompromised states. The MDR nature of *C. auris* further complicates the situation as empirical antifungal therapy frequently results in treatment failure leading to higher mortality rates. Scarcity of reliable identification and AFST facilities are among other hurdles as conventional methods usually result in misidentification. Timely institution of appropriate antifungal treatment based on reliable identification and CDC-recommended antifungal susceptibility breakpoints is crucial to decrease mortality.
